# *Mycobacterium ulcerans* Treatment Costs, Australia

**DOI:** 10.3201/eid1006.030428

**Published:** 2004-06

**Authors:** Christina Drummond, James R.G. Butler

**Affiliations:** * Monash Medical Centre, Melbourne, Victoria, Australia;; † Australian National University, Canberrra, Australian Capital Territory, Australia

**Keywords:** *Mycobacterium ulcerans*, ulcer, cost, diagnosis, treatment, case study, Australia

## Abstract

*Mycobacterium ulcerans* gives rise to severe skin ulceration that can be associated with considerable illness. The cost of diagnosis, treatment, and lost income has never been assessed in Australia. A survey of 26 confirmed cases of the disease in Victoria was undertaken. Data were collected on demographic details, diagnostic tests, treatment, time off work, and travel to obtain treatment. All costs are reported in Australian dollars in 1997–98 prices. The cost varies considerably with disease severity. For mild cases, the average direct cost is $6,803, and for severe cases $27,681. Hospitalization accounts for 61% to 90% of costs, and indirect costs amount to 24% of the total per case. *M. ulcerans* can be an expensive disease to diagnose and treat. Costs can be reduced by early diagnosis and definitive treatment. Research is needed to find cost-effective therapies for this disease.

*Mycobacterium ulcerans* causes disfiguring ulcers with substantial illness (*1*). These distinctive ulcers were first described in Australia in 1948 and given the name Bairnsdale ulcer because of their focal distribution. In Australia, in addition to the Bairnsdale area in Gippsland, Victoria, cases have been reported from foci in Queensland and the Northern Territory (*1**,**2*).

Until 1982 to 1983, when three cases were detected near Western Port Bay, cases in Victoria were confined to the Bairnsdale area. Since 1983, over 50 cases have been recorded in Victoria outside this area, and the disease appears to be spreading progressively westward. An outbreak on Phillip Island in 1993 and 1994 resulted in 27 reported cases (*3**,**4*). From 1990 to 1997, 22 cases within approximately 70 km of Melbourne were reported, 19 of these on the Mornington Peninsula with 12 in the Frankston/Langwarrin area (Figure). For the first time, cases were detected west of Melbourne in 1998, and the number occurring in that area is increasing (Victorian Mycobacterium Reference Laboratory and Department of Human Services).

**Figure:**
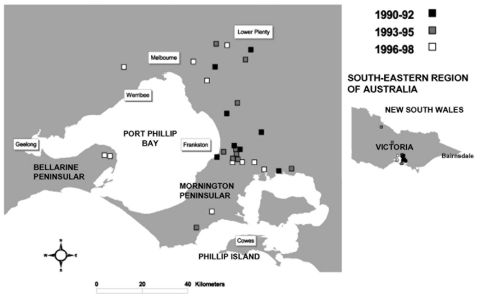
Cases of *Mycobacterium ulcerans* occurring in areas of Victoria (southeastern Australia), where disease is nonendemic, January 1990–August 1998.

Treatment involves hospitalization for debridement and often skin grafting, which frequently has to be repeated (*5**–**7*). Patients are frequently absent from work for long periods, which results in considerable cost to the community. In Australia, the cost of the diagnosis, treatment, and lost income has never been accurately assessed. The objective of this study was to assess the direct and indirect cost of *M*. *ulcerans* infection occurring outside previously known disease-endemic areas of Victoria, Australia.

## Methods

A survey of 26 cases of the disease in Victoria was undertaken. A case-patient was defined as a person with a clinical lesion with histologic, culture, or polymerase chain reaction evidence of *M. ulcerans* infection. Eligible patients with *M. ulcerans* were contacted and sent a questionnaire requesting demographic data, treatment information, time off work, and distance traveled to obtain treatment. One or more phone calls were made by the primary investigator to clarify and expand the data and seek evidence. Calls often lasted for 1 hour. Pathology reports, patient diaries, and information from physician files were used, where possible, to confirm information. The eligibility criteria were based on age (6 months of age to 100 years) and date of onset of the disease (January 1, 1991–August 31, 1998). Cases occurring in the disease-endemic areas of Gippsland and Phillip Island were excluded.

Two sources were used to find cases. The first source was the Victorian Mycobacterium Reference Laboratory. It receives specimens either directly, when mycobacteria cultures are requested, or as cultures from other laboratories to confirm mycobacterium and determine the species. The second source included records kept by a well-known Victoria pathologist with a longstanding interest in the disease; other pathologists frequently refer specimens from patients with suspected cases to this physician.

Treating physicians were contacted and approval sought from them before attempts were made to contact their patients. All known case-patients were contacted, and the questionnaire was completed by telephone interview. When a telephone number was incorrect, all leads were followed as far as possible to locate the patient. Private and public hospitals might have served as a third source for case finding, but they are not obligated to report cases of *M. ulcerans*, and their record-keeping systems are such that abstracting cases would be very difficult.

In total, 32 persons with *M. ulcerans* who met the case definition during the study period were located. Of these, 26 (81%) could be contacted. All agreed to complete the questionnaire (100% response rate).

Patients were divided into three categories on the basis of their clinical history. Patients with a mild disease had a biopsy and excision only. Patients with moderate disease had one lesion only, which was treated with biopsy and excision, with only one skin graft required. Patients with severe disease had multiple lesions, multiple debridements, or multiple skin grafts.

Medical, hospital, and other services used to diagnose and treat each patient, the time lost from his or her usual occupation, along with the cost of transportation to care facilities for patients traveling over 50 km each way were recorded. Physicians who treated the patients, hospital records, old prescriptions, and the like were used to verify cases.

### Cost Data

All cost data are expressed in Australian dollars using Australian financial year 1997–1998 prices. The unit cost of a visit to a local physician (general practitioner) and to a specialist (apart from inpatient care) was estimated by using the average fee charged across a range of relevant Medicare Benefits Schedule (MBS) item numbers for the year 1997–98 (Table 1). For specialists, the average was calculated across both first and subsequent visits. Patients treated as outpatients had a biopsy of the lesion with microscopy and culture for mycobacteria. MBS fees for the biopsy (MBS item no. 30071) and for the microscopy and culture (MBS item no. 69207) were taken as the unit costs of these tests.

**Table 1:** Cost of diagnosing and treating *Mycobacterium ulcerans* cases (Australian dollars, 1997–98 prices)^a^

Item	Unit	Cost
General practitioner visits	Visit	$24.68
Specialist visits	Visit	$57.98
Hospitalization	Cost per day for DRG 505 (other skin graft ± debridement): public hospital	$776
	Cost per day for DRG 505 (other skin graft ± debridement): private hospital	$935
	Hospitalization over 4 days	$598/day
	Bandages^b^	$18/week
	Dressings^b^	$20/week
Medications	Agent unknown	$5/course
	Clarithromycin 500 mg twice daily	$3.89/dose
	Rifampicin 600mg daily	$0.36/dose
	Ethambutol 400 mg three times/day	$0.71/dose
Home nursing	Visits	$29/visit
Laboratory costs	Biopsy (MBS item no. 30071)	$38.80
	Microscopy and culture (MBS item no. 69207)	$28.00
	Liver function tests (MBS item no. 66211)	$16.80
Heat treatment	Day^b^	$3.00/day
Lost income	Day^b^	Average weekly earnings of $766.80
Transport	Kilometer^b^	$0.50/km

^a^DRG, diagnosis-related group; MBS, Medicare Benefits Schedule. ^b^If known, the actual cost was used; otherwise, it was estimated from the unit cost.

The cost of blood tests required for monitoring a patient who was receiving special medications was estimated by using the MBS fee for liver function tests (MBS item no. 66211). Most patients receiving special medications would have these tests at least monthly.

Hospital costs were calculated according to whether the patient was treated in a public or a private hospital. For both hospital types, the per diem cost for the first 4 days of hospitalization was based on the national average cost per day for DRG 505 (other skin graft ± debridement) in 1996–97. The per diem cost for any days of hospitalization in excess of 4 was then taken as the overall average cost per day. This per diem cost is the same for both public and private hospitals (*8*).

The cost of antimicrobial agents was estimated by using the cost paid by the patient (if known) or the costs paid by a hospital pharmacy to purchase the drug. These costs were for bulk purchases of the various medications. When the name of the antimicrobial agent was unknown, the cost of a course of antimicrobial agents was estimated at $5 per course. Medications specific for mycobacteria included clarithromycin 500 mg twice daily, rifampicin 600 mg daily, and ethambutol 400 mg three times/day. Hospitals' costs for these drugs were used to estimate the cost of each patient’s course of treatment.

A visiting nurse attended the homes of some patients. The Royal District Nursing Service estimates that the average cost of a home visit was $44.10/hour (The Service, pers. comm.). When this figure is used, an average visit (including travel time) for dressing changes was estimated at 40 minutes. Thus, a rate of $29/visit was used as the estimated cost of a visit. A number of patients had heat treatment with various devices at variable costs. If known, the actual cost was included. Some patients used electrical devices such as electric blankets continuously and for prolonged periods. If costs were not known, a standard rate was used.

Dressings and bandages used by patients were priced at a commercial pharmacy, and the likely cost to the patients was calculated on the basis of their reported use. When details of the amount used were not obtainable, a standard rate for bandages and dressings was used. The cost of treatment not routinely incurred was estimated, where possible, for individual patients. These items included aids such as crutches, extra investigations such as ultrasound, occupational therapy, and physiotherapy.

The value of time lost from work because of illness was calculated by using an estimate of average weekly earnings (*9*). No cost was included for travel time per se or the time lost by children from school or kindergarten. The time lost from work by parents as a result of a child’s illness or hospitalization was also not included. No cost was included for the time lost from normal duties by retired persons or those engaged in home duties at the time of their illness. An estimate for travel expenses was included only for patients whose treatment required substantial travel (>50 km each way).

## Results

### Direct Costs

The average direct cost of treating patients with this disease was $14,608 (Table 2). This cost differed most noticeably between patients who required, or did not require, skin grafting. The average cost increased from $6,803 for patients not requiring a skin graft to $27,681 for those requiring multiple procedures.

**Table 2:** Average direct cost for diagnosing and treating a patient with *Mycobacterium ulcerans*

No. of cases	Cost in Australian dollars, 1997–98 prices (% column total)^a^			
	Mild case	Moderate case	Severe case	All cases
	12	8	6	26
Medical practitioner costs				
General practitioner visits	274	253	82	223
Specialist visits	377	739	628	546
Total visits	651 (10)	992 (6)	710 (3)	769 (5)
Other costs				
Hospitalization	4,139 (61)	12,607 (76)	24,840 (90)	11,522 (79)
Dressings	1,134 (17)	1,206 (7)	1,230 (4)	1,178 (8)
Medications	357 (5)	959 (6)	449 (2)	561 (4)
Home nursing	458 (7)	680 (4)	211 (1)	468 (3)
Laboratory costs	49 (0.7)	57 (0.3)	31 (0.1)	47 (0.3)
Heat treatment	15 (0.2)	24 (0.1)	210 (0.8)	63 (0.4)
Total direct costs	6,803 (100)	16,525 (100)	27,681 (100)	14,608 (100)

^a^Any differences between totals and sums of components are due to rounding.

Hospitalization was by far the most expensive component of the direct costs. This cost ranged from an average of $4,139 (61% of total direct cost) for mild cases to an average of $24,840 (90%) for severe cases. The second greatest expense in all categories of the disease was for dressings, approximately $1,200 for all categories of disease severity. This cost was much less than the cost of hospitalization in all categories but it was equivalent to approximately 5% of the cost of hospitalization in the severe disease category. In this category, the cost of dressings was more than double that of medications.

The third greatest expense in all categories was medical care. As the disease severity increased, the cost of medical care shifted from general practitioners to specialist care. The average cost of specialist visits ranged from $377 for mild cases to $739 for patients who received one skin graft, while average general practitioner costs fell from $274 for mild cases to $82 for severe cases. For patients with mild cases, the cost of outpatient specialist visits accounted for 58% of the cost of medical practitioner care. For patients with severe disease, this cost increased to 88%.

For the moderate and severe categories, medications were the next greatest expense, which included regular antimicrobial agents and drugs targeted for mycobacteria. These latter medications accounted for most of the cost of medications. Although approximately 50% of patients in each category were treated with these expensive drugs, those in the moderate category incurred the greatest expense as they had the longest courses of these medications. The cost per case in the moderate category was twice that in the severe category.

Only nine patients received home nursing care. One of these received 108 visits and another 96 visits. More cases in the moderate disease category received home nursing care than those in the other categories (five of the eight cases compared to two of the six in the severe category). Home nursing service in the moderate category cost an average of $680.

Heat treatment and laboratory costs were the least expensive components of the treatment costs. Heat treatment was mainly confined to the severe disease category. Laboratory costs were similar across all categories.

### Indirect Costs and Transport Costs

Time missed from regular occupation and income losses were estimated for nine patients who had regular jobs at the onset of the disease (six mild, one moderate and two severe cases) (Table 3). Of the 17 for whom income losses were not estimated, 9 were children and 5 were retired persons. Three were housewives who had difficulty estimating the amount of lost performance. A monetary value is difficult to determine for this occupation, and thus no estimate was included for their time or financial loss (Table 3). The average income loss for the nine patients increased as the severity of the disease increased, from $12,451 for those with mild cases to $16,431 and $19,170 for those with moderate and severe cases, respectively. Income losses for these nine patients were 0–$40,000. Average income losses (including for unemployed patients) are seen in Table 4.

**Table 3:** Time missed from work or school as a result of *Mycobacterium ulcerans* infection, January 1, 1991–August 31,1998

Days missed	Categorization of case (n = 23)			
	Mild (n = 11)	Moderate (n = 8)	Severe (n = 4)	All
Days missed from work or school (median)^a^	2	21	159	28
Range	0–365	0–150	0–548	0–548

^a^Some patients were retired, and some illness occurred during school holidays.

**Table 4:** Average aggregated cost per case of *Mycobacterium ulcerans* cases, January 1991–August 1998 (26 cases)

Costs	Cost in Australian dollars, 1997–98 prices (percentage of column total)^a^			
	Mild cases	Moderate case	Severe case	All cases
Total direct costs	6,803 (52)	16,525 (89)	27,681 (80)	14,608 (74)
Lost income	6,226 (47)	2,054 (11)	6,390 (18)	4,980 (25)
Transport	83 (0.6)	6 (0.0)	483 (1.4)	152 (0.8)
Total costs	13,112 (100)	18,585 (100)	34,554 (100)	19,740 (100)

^a^Any differences between totals and sums of components are due to rounding.

A small number of case-patients (n = 3) incurred major costs as a result of transportation from the country to city centers for treatment. The greatest cost estimate for this transport was $2,900 for one patient with severe disease. A patient with a mild case incurred transport costs of $1,000 and a patient with a moderate case, $46. Averaged across all patients within each severity grouping, the average transport cost per patient was $83, $6, and $483 for the mild, moderate and severe groups, respectively (Table 4).

## Discussion

*M. ulcerans* is costly to treat. This study found that the average cost of diagnosing and treating a case was $14,608. This figure is approximately seven times the average health expenditure per person in Australia in 1997–98 of $2,557 (*10*). Hospitalization costs form most of the overall costs, accounting for 90% of the total cost for severe cases and 79% for all cases. Indirect costs accounted for 25% of the overall (direct, indirect, and transportation) cost but considerably more for individual patients. For those with mild disease, income losses accounted for 47% of the overall cost.

These cost assessments are conservative for several reasons. First, most patients were treated in public hospitals as public patients; in Australia, such treatment is usually less costly than treating a private patient. Thus these costs could have been substantially higher if more cases were treated privately in Australia and could vary substantially in other populations. Second, income losses would have been much greater if more patients had been in the income-generating age group. Third, the cost of impaired productivity of parents caring for their children and accompanying them for appointments and hospitalizations was not included in this study, but this cost is likely to have been considerable.

The patients who had one skin graft incurred the highest cost for medications because they were treated with longer courses of drugs specific for mycobacteria. Three of these patients were treated for >6 months. These patients were given a trial of medication in an attempt to avert the need for surgery or as an adjunct to surgery in an attempt to prevent recurrence of the disease.

The marked decrease in general practitioner costs in severe cases reflects the referral pattern for this disease. Some patients with severe disease had previous contact with a specialist (usually a surgeon) as a result of treatment for a previous lesion, or they required more extensive surgery and skin grafting, which general practitioners would not normally perform. As patients with severe cases received more treatment as inpatients, and the cost of surgeon visits while in the hospital were included in the hospital charges, the cost of specialist treatment incurred by the patient does not clearly reflect the amount of care they received.

Variation around the mean cost within each category of disease severity was marked. Some of this variation was due to the lack of standardized, accepted treatment regimes. In addition, delays until definitive treatment varied greatly between patients, sometimes because of difficulties in making the correct diagnosis. This wide range probably accounted for the failure of indirect costs to increase in direct proportion to disease severity.

A limitation of this study was its reliance upon a patient questionnaire to collect data on diagnosis, treatment services received, and time off work. Because of the considerable amount of time that had lapsed since their illness, some patients had difficulty accurately recalling details of the events in their disease history. The number of visits to physicians and the duration of particular events such as hospitalization or time missed from work were most difficult to recall precisely. Some patients provided very accurate information because of diaries or prescriptions that they had kept during their illness or because of records from hospitals and the physicians who treated them.

Validating some information provided by patients was difficult. The date of onset, time before seeking medical help, and date of cure were frequently not documented and may be difficult to determine accurately. Where possible, attempts were made to validate dates and number of physician visits or duration of hospitalization, but insufficient resources were available to validate all data provided by patients. A prospective study, performed at the time of an outbreak of this disease, would be useful to more accurately determine the cost of treatment.

The early diagnosis and implementation of effective, definitive treatment would greatly reduce both the illness and economic impact from *M. ulcerans* infection. Education of medical practitioners and the public is required. If community awareness of this disease is increased, patients will seek treatment earlier. Educating medical practitioners about the clinical features of this disease and its recent geographic spread are essential for early diagnosis and appropriate treatment to be promptly implemented. Since this disease is very appropriate for nursing assistance at home, hospitalization costs can be drastically reduced by implementing this method of treatment after surgery. Early and definitive treatment would also substantially reduce the time missed from work and the associated cost.

The role of medication directed against mycobacteria, heat therapy, and other therapies reported as helpful by individual patients requires investigation. An alternative to the extensive surgery currently required by most patients would greatly reduce the illness and cost of this disease. Recently, much progress has been made in detecting these bacteria in the environment and in rapidly diagnosing lesions (*11**,**12*).

*M. ulcerans* is of increasing public health concern worldwide. In African countries, treatment costs for one case far exceed the governmental health spending per capita (*13*). The increasing numbers of cases in many countries, the increasing number of countries affected, and the substantial disability and loss of income that result from this disease underscore the need for continuing research into rapid diagnostic methods and cost-effective treatments.
